# Data-based program management of system-wide IV smart pump integration

**DOI:** 10.1093/ajhp/zxad245

**Published:** 2023-10-07

**Authors:** Karen K Giuliano, Rebecca S Mahuren, Jacob Balyeat

**Affiliations:** Elaine Marieb Center for Nursing and Engineering Innovation, University of Massachusetts Amherst, Amherst, MA, USA; Parkview Health, Coldwater, MI, USA; Parkview Regional Medical Center, Fort Wayne, IN, USA

**Keywords:** auto-documentation, auto-programming, health information interoperability, infusion pumps, medication error, patient safety

## Abstract

**Purpose:**

Smart pump bidirectional interoperability offers automated infusion programming and documentation that can improve patient safety and workflow efficiency. This technology has been poorly implemented across US hospitals, and there is little guidance on the tracking or monitoring of interoperability systems. The purpose of this report is to describe the successful implementation of intravenous (IV) smart pump interoperability in a large health system.

**Summary:**

Bidirectional IV smart pump interoperability and compliance monitoring were implemented across a large Midwestern health system using ICU Medical’s Plum 360 and LifeCare PCA devices and Smith Medical’s MedFusion 4000 Syringe Pump devices. The hospital system’s experience in implementing and monitoring IV smart pump compliance using automated reports and a dedicated medication safety integration nurse is described. Compliance trends suggest that the implementation of IV smart pump interoperability has achieved a reduction in programming outside of the dose error reduction system, manual overrides, and IV medication administration error rates.

**Conclusion:**

The monitoring of smart pump compliance has had demonstrated benefits in investigating usability concerns, recognizing system errors, and identifying increased needs for nurse training. This program can serve as an example for other healthcare systems adopting IV smart pump interoperability.

**Key Points**

- Bidirectional IV smart pump interoperability was successfully implemented in a large Midwestern health system with the creation of a pump compliance tracking and monitoring program.
- The capacity to continuously monitor IV smart pump compliance has demonstrated benefits in identifying usability concerns, recognizing system errors, and identifying increased needs for nurse training.
- A multidisciplinary team with a dedicated medication safety integration nurse was critical to successful IV pump interoperability implementation, ongoing compliance monitoring and tracking, and rapid resolution of usability and compliance issues.

Traditional intravenous (IV) smart pumps with drug libraries and a dose error reduction system (DERS) were first introduced into clinical use in 1996.^1^ Unfortunately, the manual use of IV smart pumps has not been associated with a measurable decrease in adverse drug events.^1-4^ IV smart pump interoperability automates the process and offers additional safety benefits by making medication errors recognizable and rectifiable.^5^ Many healthcare systems in the United States have implemented technologies required for interoperability, such as computerized prescriber order entry, barcode-assisted medication administration, and electronic health records (EHRs), yet most hospitals have not integrated these systems with IV smart pumps to achieve bidirectional interoperability.^6-10^ Broad implementation of bidirectional IV smart pump interoperability could improve patient safety, clinical outcomes, and work efficiency.^3,7,11-14^

## Bidirectional interoperability

Bidirectional IV smart pump interoperability involves 2-way, real-time, continuous communication between a smart pump and the EHR that enables both auto-programming and auto-documentation.^6,15^ The goals of using bidirectional smart pump interoperability are to improve workflow at the point of care and decrease IV infusion–related medication errors and infusion-related adverse drug events.^15,16^ Consistent use of bidirectional IV infusion systems reduces the need for manual data entry, although manual entry is still needed in emergent situations, such as “code blue” emergencies, trauma, rapid response team calls, and system downtimes. Recognizing its benefits to patient safety, in 2020 the Institute for Safe Medication Practices (ISMP) recommended that institutions using IV smart pumps implement bidirectional auto-programming and auto-documentation interoperability with their EHRs.^11^

Auto-programming occurs when a barcode is scanned and triggers the transmission of the provider-ordered, pharmacist-approved infusion data from the EHR to a specific channel on the IV smart pump. The infusion parameters are then prepopulated and displayed on the IV smart pump display screen for clinician review and acceptance. Auto-programming reduces the manual keystrokes of all IV administrations by approximately 86%,^11,12^ has a demonstrated benefit in reducing programming errors,^14^ increases DERS compliance, and decreases the number of triggered DERS alerts.^13^ However, even when implemented, auto-programming faces challenges with clinician compliance related to a steep learning curve, reluctance to adopt new workflows, time constraints, and training requirements.^14^

Auto-documentation, also known as auto-charting or infusion documentation, occurs when the time-coded infusion data are wirelessly sent from the IV smart pump back to the EHR for clinician review and acceptance.^11^ Infusion data includes the medication name, dosage, volume infused, and infusion duration.^8^ More importantly, auto-documentation captures infusion data that is often missing from documentation, including rate and dose changes and exact stop times.^11^ Thus, auto-documentation decreases inconsistencies in clinician documentation and increases EHR completion rates.^9^ Timely capture of infusion therapy details also reduces adverse events and improves billing accuracy, which can increase hospital revenue.^17^

Because the vast majority of IV smart pumps in current clinical use were not designed with bidirectional interoperability in mind, technical hurdles must be addressed before implementation, including connecting siloed technologies^6,15^; maintaining a complex, facility-specific drug library; and addressing significant usability issues. These usability issues include the need for advanced, accurate, and scalable informatics technologies (eg, EHR, smart pumps, safety software), standardized clinical workflow practices including bedside equipment and room layout support, standardized drug dosages and protocols, robust wireless infrastructures, a method for tracking compliance with the DERS to support the ISMP best practice goal of at least 95% compliance, and additional personnel to implement and maintain all aspects of these systems.^5,15,16,18,19^

Finally, systematic approaches are needed to track and monitor IV smart pump interoperability.^7^ Analyzing interoperability systems requires specific knowledge and expertise. Currently, there are no specific standards for the evaluation of IV smart pump interoperability. Additional barriers include software event logs that are poorly designed for the analysis of incident investigations^20^ and insufficient dedicated personnel to evaluate infusion analytics.^7^ The purpose of this medical technology report is to share our experience in creating and using automated IV smart pump compliance reports to continually monitor and evaluate the safety, effectiveness, and usability of an IV smart pump interoperability and integration program.

## Program description

### Device selection.

This program was initiated at Parkview Health, a large, 11-hospital health system in the Midwest that uses 3 integrated IV smart pumps from 2 different vendors. The Plum 360 smart pump (ICU Medical, San Clemente, CA) was implemented in November 2017, and ICU Medical’s LifeCare PCA was implemented in February 2020. The MedFusion 4000 Syringe Pump (Smith Medical, Minneapolis, MN) was implemented in March 2021. Each of these smart pumps has a different user interface. The Plum 360 compliance report was selected to monitor overall program quality for all 3 smart pumps due to its capacity to show clear trends over time.

### Planning.

This program required 2 distinct stages of planning and implementation: (1) the deployment of each of the IV smart pumps with the associated IV smart pump compliance monitoring and (2) the implementation of IV smart pump bidirectional interoperability with the EHR (Epic Systems Corporation, Verona, WI).

**Figure 1.**
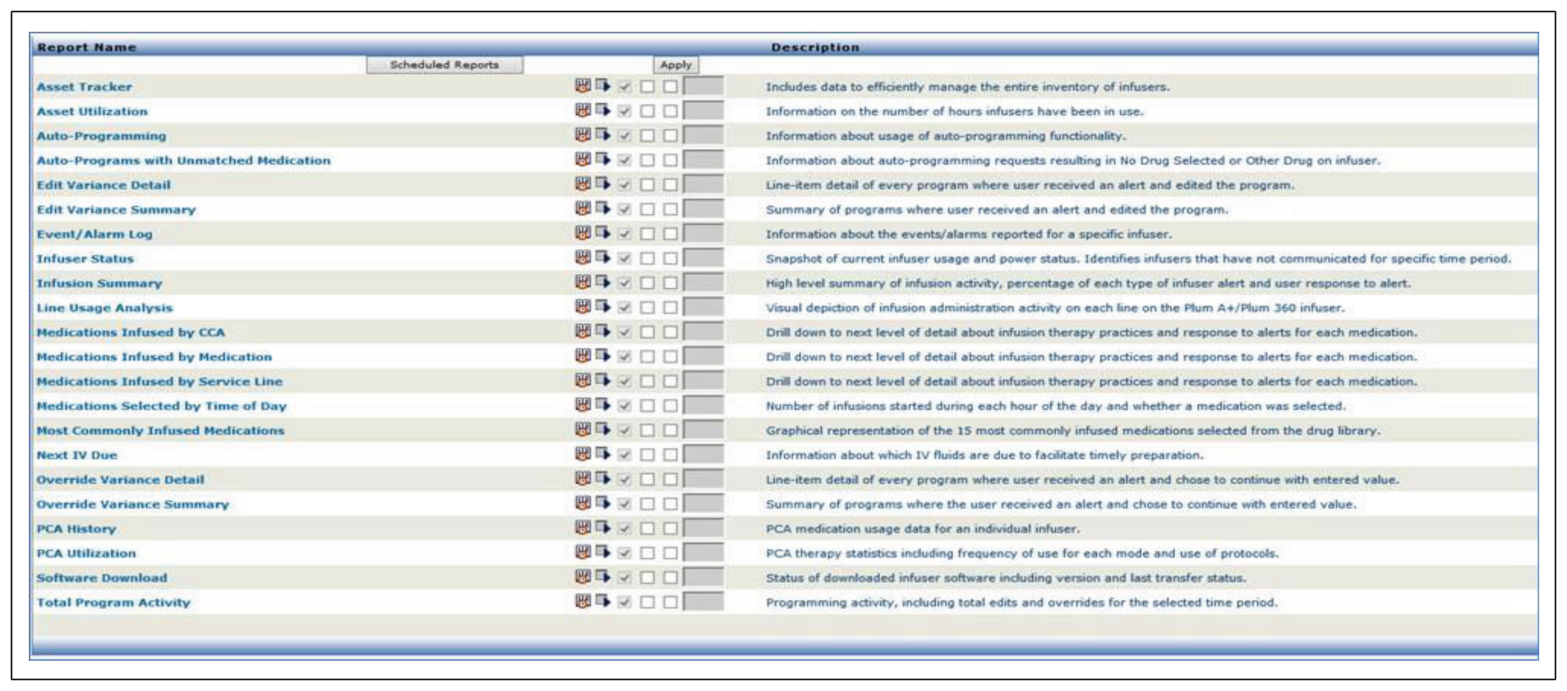
Screenshot of available MedNet reports for ICU Medical smart pump program management.

Multiple automated reports were designed by an interdisciplinary team of nursing, pharmacy, biomedical, and information technology (IT) personnel to facilitate continuous tracking and monitoring of IV smart pump compliance. Customized reports were created to automatically track and compile IV smart pump medication data. These data included medication and fluid administration via an IV smart pump channel, infusion documentation, the number of IV infusions, number of patient admissions, nurse compliance percentage for use of the IV smart pump DERS, and medication administration error rate. Figure 1 shows a list of the types of standard vendor-created reports used to manage the program. Reports were also created from the EHR database to identify workflow and usability issues. These reports compiled data about user compliance, number of attempts to auto-program, and programming error types.

**Figure 2.**
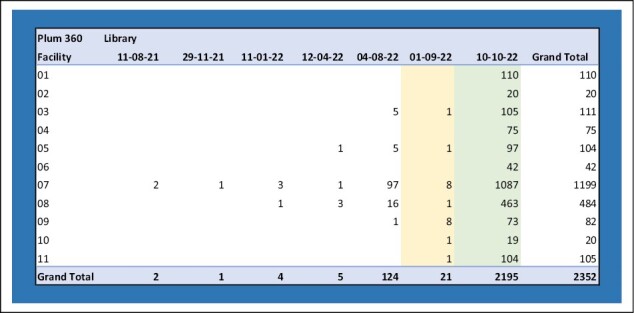
Example of ICU Medical Plum 360 system BioMed report for tracking the timeliness of drug library updates.

The IV smart pump compliance reports allow for real-time data analysis at various levels of granularity, including by hospital, clinical unit, or individual clinician. Figure 2 shows an example of a Plum 360 system BioMed report to track the timeliness of drug library updates, which allows the user to drill down for additional detail.

The Parkview Health medication safety committee determines what data is preferred for monthly review (eg, Infusion Summary, Edit Variance Detail, and Override Variance Detail). These reports have assisted with identifying medications requiring further investigation (for example, heparin infusions programmed above the upper hard limit, requiring system edits). The Event/Alarm Log report is used to review pump activities—manual and auto-programming—following a medication event. The other reports are used to investigate infusion activities prior to order set updates or special requests by clinical specialists.

The second stage of this program was the implementation of IV smart pump bidirectional interoperability. Auto-programming and auto-documentation capabilities were achieved by integrating the health system’s drug library and IV smart pumps. Three server environments were created in preparation for this integration. Two nonproduction environments were created to build and test the initial drug library before its deployment in real clinical settings. One nonproduction environment was later repurposed to function as an ongoing educational platform server for new-hire user training, while the other continues to be used for new builds and testing prior to deploying any major changes into the live clinical system. The third server environment is the live environment.

Parkview Health has been using the Plum 360 legacy pumps since 2009, so staff are very familiar with the manual operation of the pumps. Nurses were trained on IV smart pump interoperability through a 2-hour classroom session that included (1) a step-by-step presentation and (2) a 2-minute video demonstration, followed by (3) hands-on simulations of auto-programming of various infusion workflows, including primary and secondary infusions, titrations, rate changes, and auto-documentation. Use of this real-world, hands-on simulation training for nurses is key for the success of the program. Other clinical providers and prescribers who do not actually operate the pumps received an SBAR (situation, background, assessment, recommendation) overview of interoperability and regular updates to flyers regarding drug library changes and limitations.

### Implementation.

**Figure 3.**
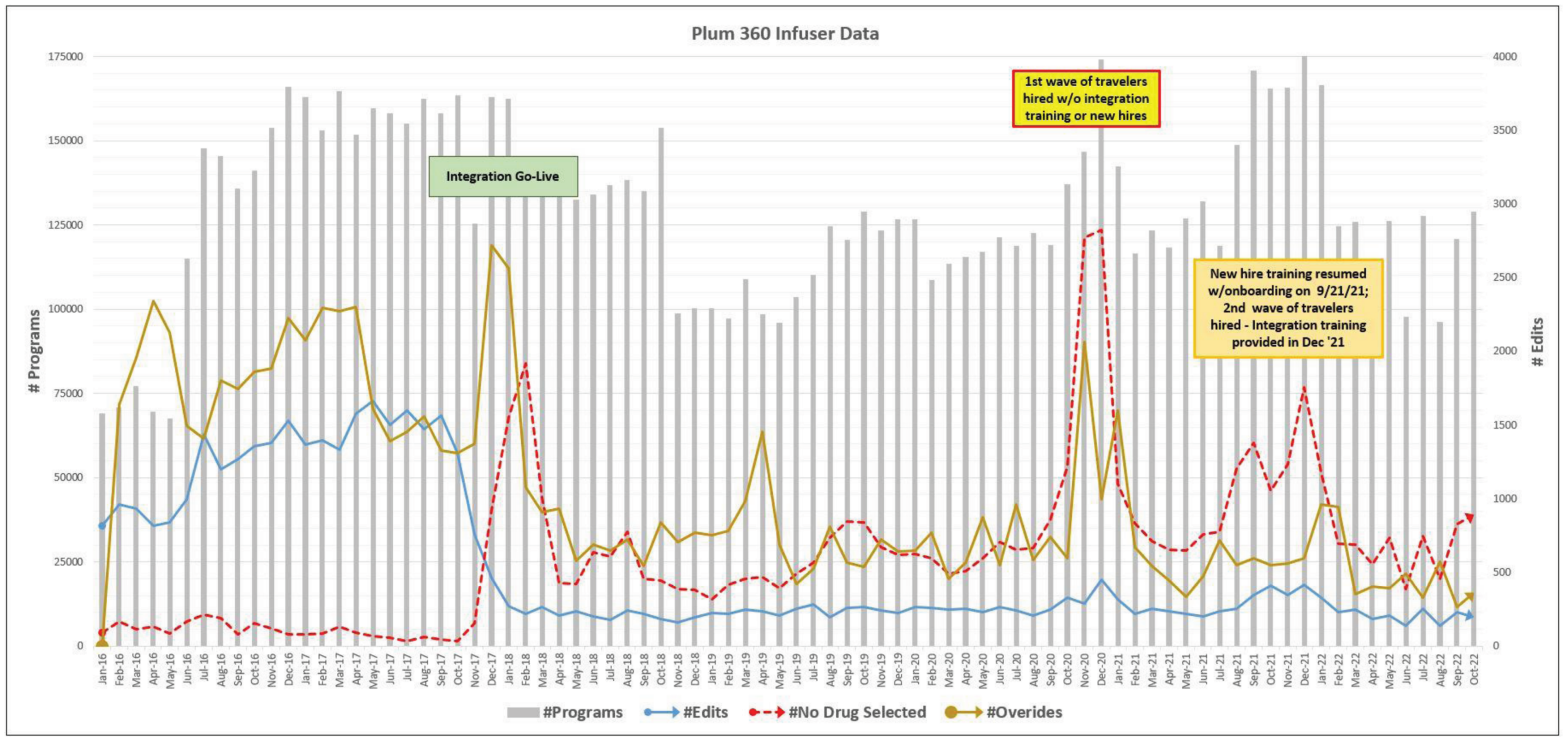
ICU Medical Plum 360 report highlighting 2 major time periods where increases in programming outside of the dose error reduction system, manual overrides, and edits occurred.

As shown in Figure 3, initiation of IV smart pump monitoring using customized reports began in May 2016 with the deployment of the Plum 360 IV pumps throughout the health system. This was followed by integration of the IV smart pumps with the drug libraries in November 2017. Data from the reports are focused primarily on the Plum 360 large-volume pumps. Planning for each of the stages of implementation took several months. Throughout the 11-hospital system, there are 3,500 Plum 360 large-volume pumps, as compared to 130 LifeCare PCA (patient-controlled analgesia) pumps, and 230 MedFusion 4000 Syringe Pumps. An experienced, system-level medication safety integration nurse collaborated closely with clinical specialist pharmacists to define medication settings and participate in the review process in preparation for drug library and pump integration. Tracking and monitoring of IV smart pumps continues to be actively led by this medication safety integration nurse.

Data from the customized reports are generated monthly by the medication safety integration nurse to analyze and review for overall system use and to identify any trends that may be of concern. Examples of data specifically reviewed in the monthly reports include drug library compliance and safety alert overrides. These data analyses are also provided to the multidisciplinary medication safety committee each month for their review. Data are used as needed to inform changes in the drug libraries, clinical workflows, or EHR integration to support ongoing system optimization. Reports are also generated and reviewed any time staff report concerns that can be addressed using pump data. Finally, ICU Medical, the vendor for the Plum 360 IV pump, has assigned a pharmacist within that organization to analyze pump data and communicate findings and recommendations for system optimization.

Support from the hospital system’s administration has been key in the successful integration implementation projects. For example, when we first enabled interoperability, unforeseen server settings and cached auto-programming messages prevented integration from successfully reaching the pump or the medication administration record. Following an in-depth collaborative investigation with the vendors, which was supported by the hospital system administration, we updated the interoperability implementation strategy to occur incrementally by departments. This revised approach allowed IT to better monitor integration messages and correct any issues before going live with additional departments. Administration also supported the creation and training of additional super-user nurses at the point of care to provide ongoing staff support and system troubleshooting.

In addition to having nurse super-users, we have created pump training video clips that are accessible at the point of care by QR codes. The codes are affixed to each pump and offer the opportunity for additional hands-on training anytime it is needed. Finally, there are pump training stations at the profession skills education days and a mobile training station that can be brought to the clinical units as needed to provide additional hands-on supervised practice. As a result of this level of support, the integration of the IV smart pumps was well received, and even celebrated, by the nursing staff.

## Program experience

### Drug library updates.

A critical component of the programs’ success is the ability to provide efficient drug library updates any time they are needed. Parkview Health has an intranet request ticket for updates (eg, new drug, changes or deletion) for medications for infusion pumps. The requests are reviewed by the pharmacy and therapeutics committee along with all new infusions and special infusions requested by physicians.

Prior to deploying drug library updates to the live clinical system, they are first deployed and tested in the training system. This allows us to address any unexpected issues prior to use on actual patients. Once testing is complete, drug library updates are deployed to the pump fleet and monitored. Typically, within 72 hours 75% of the fleet has been successfully updated. Prompt updates are important to minimize the availability of different versions of the drug library being used simultaneously in patient care. Difficult-to-locate pumps, which may be tucked into a storage unit, on emergency medical services trucks, or moved off-site, are located and updated as quickly as possible.

Prior to the interoperability implementation, drug library updates occurred quarterly. With use of interoperability to auto-program and document infusion activities, the frequency of drug library updates increased to every other month. Only during the COVID-19 pandemic and the emergency use authorization by the US Food and Drug Administration did library updates occur more frequently.

### Implementation challenges.

**Figure 4.**
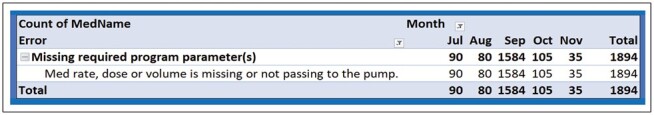
Report highlighting a large decrease in auto-population of the rate field and auto-programming.

***Case example.*** At one point, staff reported that suddenly EHR orders no longer auto-populated the rate field on the Plum 360 pumps, causing auto-programming to fail. Initial inquires of recent pharmacy information system and EHR upgrades that may have impacted integration were dismissed until pump data showed 80 to 90 instances of auto-programming failure during the previous 2 months and, in the current month, over 900 instances in the first 2 weeks (Figure 4). These supporting data prompted a deeper dive, and the investigation revealed that the cause was, indeed, the recent upgrades. As a result, a fix was quickly designed and tested in the nonproduction system and then migrated into the live system—something that would not have occurred as quickly without ready access to the smart pump data reports. Once the fix was complete, continued monitoring using the reports confirmed that the rate field auto-population and auto-programming capability was again functioning properly.

#### Integration of existing information.

Analysis of IV smart pump compliance and usability must consider the context and complexity of the health system. Trend data for Parkview Health (Figure 3) showed evidence of 2 major time periods where increases in programming outside of the DERS, manual overrides, and IV medication administration error rates occurred. The first period was during initial program implementation, and the second was during the COVID-19 pandemic, coinciding with the arrival of both traveling nurses and new IV therapies not yet built into the drug library. These trends underscored the importance of nurse training, the need for a comprehensive drug library that meets the clinical needs of different patient populations, the importance of planning when there are major shifts in the delivery of care, and the need for ongoing monitoring at the system level in order to recognize and better understand changes in IV smart pump use and how these changes may impact both nursing workflow and patient care.

#### Limitations.

Interoperability has not been implemented in procedural and perioperative areas, including anesthesia, endoscopy, cardiac catheterization laboratory, dialysis, and interventional radiology areas. Interoperability is also not used during certain clinical patient care scenarios, such as emergency transport between facilities, trauma resuscitation, code blue/cardiac resuscitation events, rapid response team calls, administration of blood products, and rapid fluid resuscitation (ie, situations involving infusion rates of >999 mL/h, gravity infusions, and pressure bag–administered infusions).

## Discussion

This program provided insight into the benefits of customized reports for system-level monitoring of IV smart pump use when implementing IV smart pump interoperability. It also provided a real-world example of successful integration when using IV smart pumps from different manufacturers and the strengths of continuous IV smart pump monitoring across a large health system. An assessment of IV smart pump compliance and usability before and after drug library integration in November 2017 demonstrated an overall reduction in manual overrides, programming outside of the DERS, and IV medication administration error rates (Figure 3). Average annual rates of manual overrides decreased from 2.6% to 0.36% from before program implementation to October 2022; average annual rates of programming edits decreased from 1.27 per 100 programs to 0.18 per 100 programs over the same time.

The capacity to continuously monitor IV smart pump compliance has had benefits in terms of (1) identifying usability concerns, (2) recognizing system errors, and (3) identifying increased needs for nurse training. As shown in Figure 3, while the influx of new, untrained nurses resulted in a temporary increase in noncompliance and infusion errors, program monitoring at the system level was able to identify the trends, leading to the development of a targeted mitigation and improved drug library compliance.

Compliance monitoring has enabled the rapid recognition of and response to inadequate nurse training at the individual nurse level. For example, several individual users who were consistently programming outside of the DERS were identified and re-educated. Overall system trends revealed increased noncompliance and infusion errors during periods of new medications and staff, which is what led to investment in the development of the hands-on IV smart pump integration training module for onboarding nursing staff, including traveling nurses, that was previously described. Use of this training system for training all new staff or retraining current staff has improved compliance and decreased errors. In addition, a point-of-care IV smart pump training resource that can be accessed using a QR code has also been attached to all IV smart pumps, which provides immediate access to a “how to” video clip playlist that can be used by clinicians when and where it is needed.

In our experience, the multidisciplinary approach was the most critical component of the program’s success. Close collaboration with all the hospital stakeholders, including IT, biomedical engineering, nursing, and pharmacy personnel, was essential to plan the implementation, develop monitoring capabilities and IT reports, quickly recognize when things were working, and minimize program delays. The EHR vendor determined the specific technical interoperability implementation requirements. Fortunately, Parkview Health had only one drug formulary for the entire health system, which allowed the EHR and pump teams to focus on one drug library and greatly simplified the entire process.

Another critical component of success was the use of a dedicated medication safety integration nurse who is an expert in the system implemented. The medication safety integration nurse is available to address the needs of various stakeholders, especially end-user nursing staff, so that issues can be identified and addressed as quickly as possible. Rapid resolution of usability issues at the point of care is fundamental for maintaining nursing staff confidence in the technology and preventing nurses from reverting back to manual programming and system workarounds when uncertainly arises.

## Conclusion

Bidirectional IV smart pump interoperability offers great potential for improving patient care, clinical workflows, and IV medication administration accuracy and safety. In addition to the use of standardized tracking and monitoring, improvements in IV smart pump technology are needed in order to increase the adoption of bidirectional IV smart pump interoperability in US healthcare. We have reported our own experience with the implementation of IV smart pump interoperability and continuous tracking using automated compliance reports to illustrate a possible solution and its required resources. This program implementation resulted in measurable improvements in IV smart pump compliance and IV medication administration error metrics. We have also demonstrated the advantages of continuous interoperability tracking, including the ability to identify usability concerns, recognize system errors, and promptly respond to insufficient nurse training. Other healthcare systems may consider establishing similar programs to increase IV smart pump safety.

## Data Availability

The data underlying this article are available in the article.
